# Differentiating Nontuberculous Mycobacterial Lung Disease from Pulmonary Tuberculosis: A Retrospective Study on Clinical Features and a Diagnostic Model

**DOI:** 10.3390/jcm15176796

**Published:** 2026-09-02

**Authors:** Yuanjie Li, Shuang Zhao, Jinyan Li, Jing Ren, Yuan Wang, Yaoxi Chen, Chuanqi Wei, Xiaofeng Xiong, Zhixin Qiu

**Affiliations:** 1 Department of Pulmonary and Critical Care Medicine, West China Hospital, Sichuan University, Chengdu 610065, China; 2 State Key Laboratory of Respiratory Health and Multimorbidity, West China Hospital, Sichuan University, Chengdu 610041, China; 3 The Integrated Care Management Center, West China Hospital, Sichuan University, Chengdu 610041, China

**Keywords:** nontuberculous mycobacterial pulmonary disease, pulmonary tuberculosis, differential diagnosis, predictive model

## Abstract

**Background**: Nontuberculous mycobacterial pulmonary disease (NTM-PD) and pulmonary tuberculosis (PTB) present with similar clinical and radiological features, frequently leading to misdiagnosis. This study aimed to compare NTM-PD with extrapulmonary NTM disease (ENTM) and PTB and to develop a predictive model for early differentiation. **Methods**: We retrospectively analyzed 473 NTM patients (340 NTM-PD, 112 ENTM, 21 disseminated) and 218 PTB patients treated between January 2008 and December 2024. A diagnostic model was developed using the Least Absolute Shrinkage and Selection Operator (LASSO) regression algorithm and multivariate logistic regression, with internal validation via 1000 bootstrap resamples and a pre-split validation cohort. Performance was evaluated by the area under the receiver operating characteristic curve (AUC), calibration curves, and decision curve analysis. **Results**: Compared to ENTM, NTM-PD patients were older and had lower BMI, with bronchiectasis (OR = 24.86) and prior PTB history (OR = 7.58) as the strongest risk factors. Relative to PTB, NTM-PD patients were more often female and older, with more bronchiectasis and mucus plugs on imaging, while PTB showed more pleural thickening and effusion. NTM isolates exhibited high resistance to first-line anti-TB drugs but maintained susceptibility to macrolides and amikacin. The nine-predictor nomogram achieved AUCs of 0.758 (training) and 0.731 (validation), with calibration and decision curve analysis confirming clinical utility. **Conclusions**: NTM-PD and PTB exhibit distinct clinical, radiological, and microbiological profiles. The nomogram offers a useful preliminary screening tool for early differentiation, though external validation in multicenter studies is warranted.

## 1. Introduction

Nontuberculous mycobacteria (NTM) infections have emerged as increasingly significant opportunistic pathogens, with a steadily rising global incidence and detection rate [1,2,3]. A recent study conducted in the United States confirmed this trend, reporting that the average annual incidence of NTM among the Medicare population reached 30.4 per 100,000 between 2010 and 2019, with a steady increase from 24.4 to 36.7 per 100,000 over the decade [4]. Similarly, data from a South Korean study revealed a marked increase in both the prevalence (from 1.2 to 33.3 per 100,000 population) and incidence (from 1.0 to 17.9 per 100,000 population) of NTM infections between 2003 and 2016 [5]. Among the diverse NTM pathogens, *Mycobacterium abscessus* (*M. abscessus*) and the *Mycobacterium avium-intracellulare* complex (MAC) are the most prevalent. These infections can manifest in both pulmonary and extrapulmonary sites, with pulmonary involvement accounting for the vast majority—over 90% of cases [6]. However, the epidemiological characteristics of patients with infections at different sites remain poorly understood.

As the primary clinical manifestation of NTM infection, the incidence and prevalence of NTM pulmonary diseases (NTM-PD) are steadily increasing worldwide [7,8]. Its clinical and imaging features significantly overlap with those of pulmonary tuberculosis (PTB), frequently leading to misdiagnosis, particularly in countries with a high TB burden [9,10]. Inappropriate anti-tuberculosis treatment can cause adverse effects and delay appropriate management, while reliance on time-consuming traditional culture and subsequent identification further prolongs the diagnostic process [11,12]. Therefore, a deeper understanding of the clinical distinctions between the two diseases and the development of early differential diagnostic models are critically important.

Based on the research background and clinical diagnostic challenges, this study aims to systematically analyze the epidemiological and clinical characteristics of NTM patients with different sites of infection. By developing an early differential diagnostic model, we seek to improve the distinction between NTM-PD and PTB, thereby providing evidence to support precise clinical interventions.

## 2. Materials and Methods

**Figure 1:**
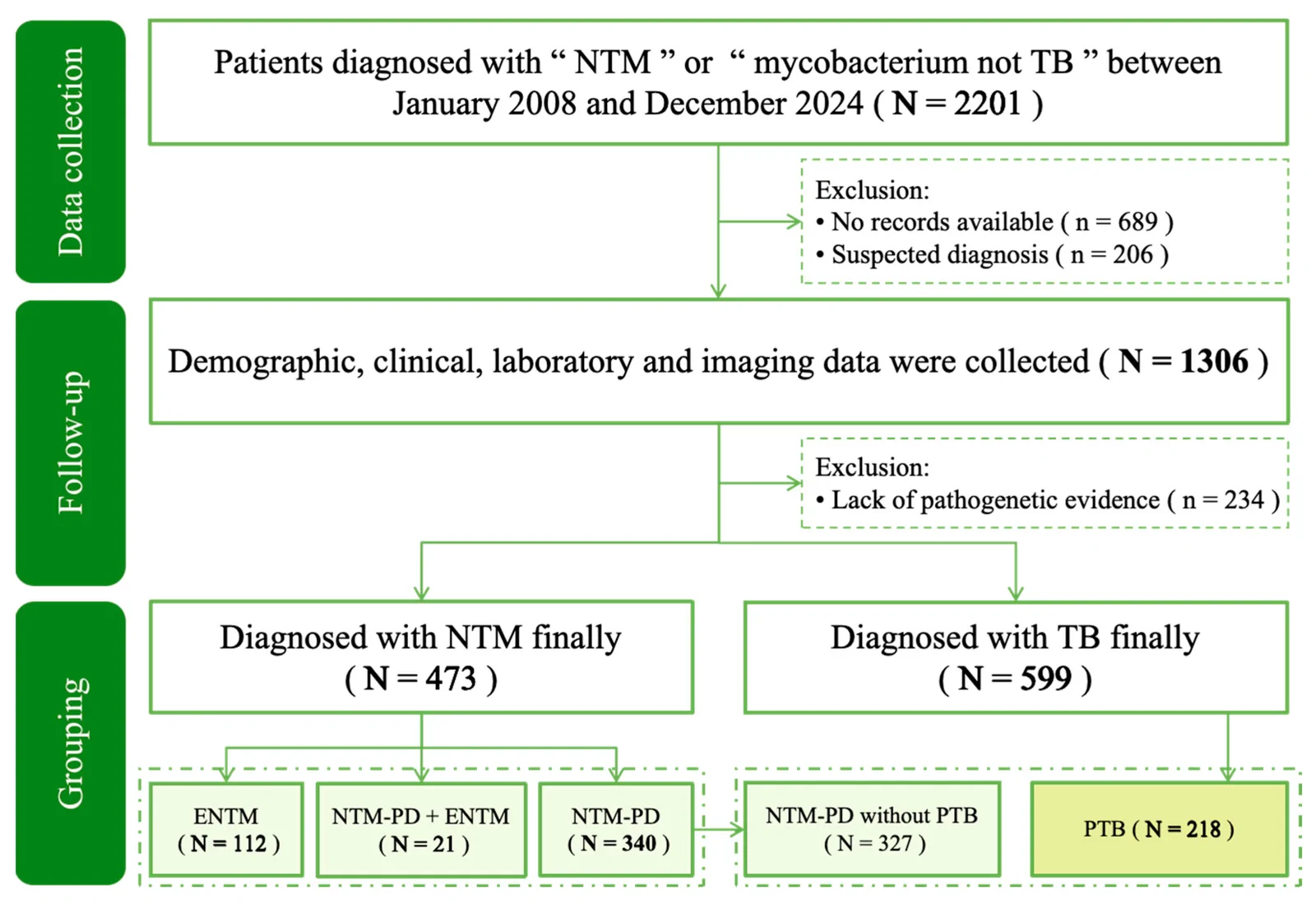
Flowchart for the selection of the study population. Abbreviations: NTM: nontuberculous mycobacteria; TB: tuberculosis; ENTM: extrapulmonary nontuberculous mycobacteria diseases; NTM-PD: nontuberculous mycobacterial pulmonary diseases; PTB: pulmonary tuberculosis.

A retrospective analysis was conducted on patients suspected of NTM infections at West China Hospital of Sichuan University between January 2008 and December 2024 (Figure 1). Inclusion criteria included documented NTM infections at any anatomical site, adherence to the 2020 ATS/IDSA diagnostic criteria, and definitive microbiological confirmation of NTM presence [13]. The clinical diagnosis of PTB was consistent with WHO guidelines [14]. Exclusion criteria were (1) incomplete clinical or medical history records and (2) indeterminate or unconfirmed microbiological diagnosis; A total of 1129 patients were excluded during screening, resulting in 473 patients with NTM infections and 599 patients with TB included in the final analysis. The modeling cohort ultimately included 327 patients with pure NTM-PD and 218 patients with PTB, while 13 cases of co-infection with NTM and TB were excluded. The rationale for exclusion was that co-infection could confound the clinical and radiological features attributed to NTM, making it difficult to determine whether the observed characteristics were due to NTM, TB, or their combined effects. This study was supported by the Ethics Committee, with a waiver of informed consent.

In this study, two investigators collected patients’ demographic characteristics, clinical symptoms, laboratory test results, treatments, and outcomes. Two independent pulmonologists evaluated the imaging features, and any discrepancies in diagnosis or assessment were resolved by a third respiratory specialist. The primary data collected included symptoms, imaging features, laboratory test parameters, pathogens, and drug susceptibility test results.

Clinical specimens were pretreated using standard N-acetyl-L-cysteine/NaOH decontamination and then cultured using both the automated BD BACTEC™ MGIT™ 960 system (Mycobacteria Growth Indicator Tube; Becton, Dickinson and Company, Franklin Lakes, NJ, USA) and conventional Löwenstein-Jensen slants until visible growth was detected. Species identification from positive cultures was performed using matrix-assisted laser desorption/ionization time-of-flight mass spectrometry (MALDI-TOF MS) [15]. Minimum inhibitory concentration (MIC) values for antimicrobial agents were determined using Mycobacterium Drug Susceptibility Löwenstein-Jensen Medium (Yinke, Zhuhai, China). Anti-mycobacterial susceptibility was assessed based on MIC breakpoints, following the Clinical and Laboratory Standards Institute (CLSI) M24S 2018 guidelines.

Clinical characteristics were summarized descriptively. Continuous variables are reported as mean ± SD or median (IQR) based on distribution normality; Categorical variables are expressed as frequencies and percentages. Between-group comparisons for categorical variables were conducted using the chi-square test or Fisher’s exact test when expected counts were less than 5. A two-sided *p*-value of less than 0.05 was considered statistically significant. All analyses were conducted using R version 4.3.3.

Eligible patients were randomly divided into a training cohort and an internal validation cohort at a 2:1 ratio using stratified randomization based on diagnosis (NTM-PD vs. PTB) to ensure comparable outcome prevalence between datasets. To develop a robust diagnostic model, we first performed feature selection using the Least Absolute Shrinkage and Selection Operator (LASSO) regression algorithm with 10-fold cross-validation. The optimal penalty parameter λ was selected as the largest value within one standard error of the minimum cross-validated deviance. Variables with non-zero coefficients were retained as candidate predictors and subsequently entered into a multivariable logistic regression model using backward stepwise selection based on the Akaike Information Criterion (AIC), with variables achieving *p* < 0.05 incorporated into the final nomogram. To rigorously assess model stability and generalizability while mitigating overfitting concerns—particularly given the relatively large number of initial candidate variables—we performed internal validation using two complementary approaches: (1) 1000 bootstrap resamples drawn from the training cohort to calculate optimism-corrected performance metrics (including AUC and C-index), and (2) direct evaluation of the final model in the pre-split validation cohort to provide an unbiased estimate of discriminative ability. Model discrimination was quantified using the area under the receiver operating characteristic curve (AUC) and Harrell’s C-index, while calibration was assessed via calibration plots with the Hosmer-Lemeshow goodness-of-fit test. Decision curve analysis (DCA) was performed to evaluate clinical net benefit. All statistical analyses were conducted using R software (version 4.3.3), and a two-sided *p* < 0.05 was considered statistically significant.

## 3. Results

### 3.1. Baseline Characteristics

**Figure 2:**
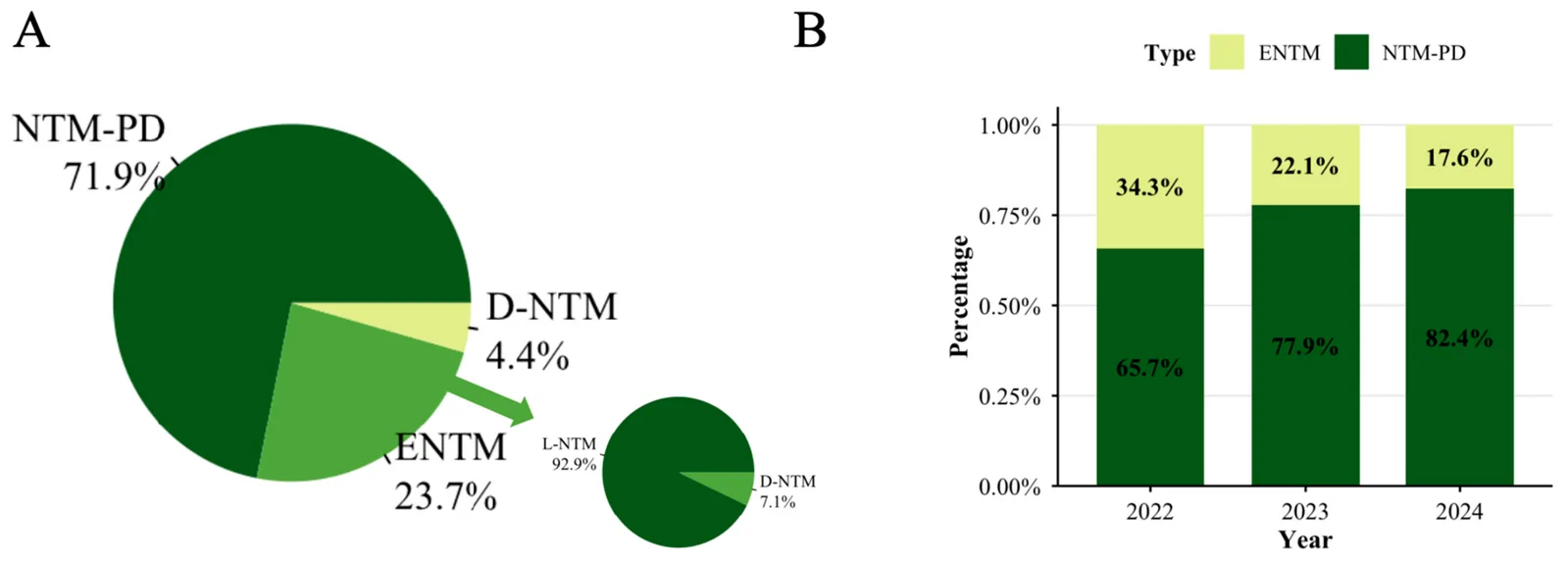

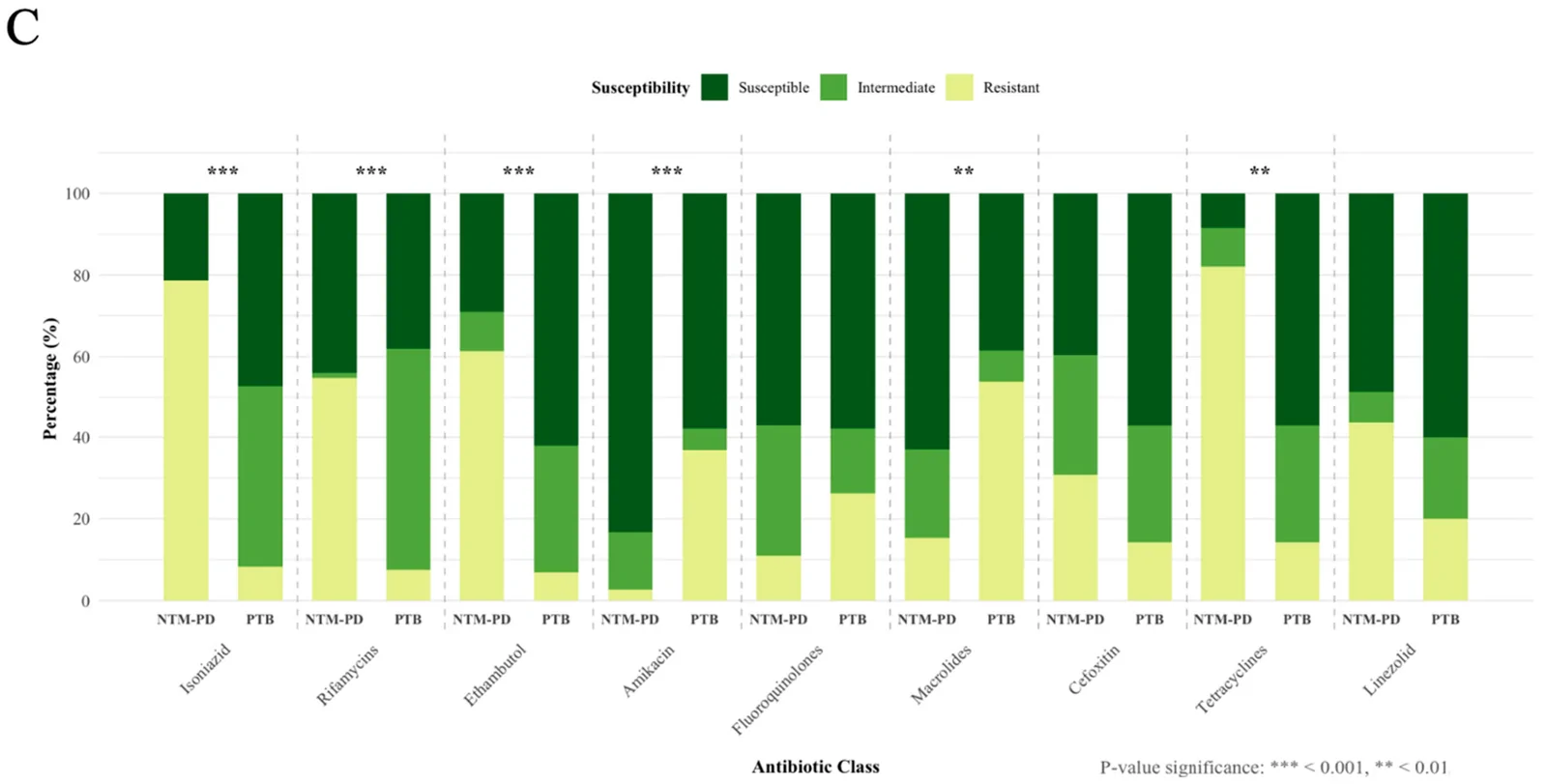
Distribution and temporal patterns of NTM infections. (**A**) NTM infection type distribution. (**B**) Temporal changes in NTM infection type distribution (2022–2024). (**C**) Antibiotic Susceptibility Profile (Percentage) with Statistical Significance. Abbreviations: NTM: nontuberculous mycobacteria; ENTM: extrapulmonary nontuberculous mycobacteria diseases; NTM-PD: nontuberculous mycobacterial pulmonary diseases; L-NTM: localized NTM; D-NTM: disseminated NTM; PTB: pulmonary tuberculosis.

Ultimately, we identified 473 patients with NTM infection, including 340 (71.9%) cases of NTM-PD, 112 (23.7%) cases of extrapulmonary NTM (ENTM), and 21 (4.4%) cases with concurrent pulmonary and extrapulmonary infections (Figure 2). Over the past three years, the proportional distribution of NTM-PD has shown a year-on-year increase compared to ENTM (Figure 2). Among these patients, 267 (56.4%) were female, with a median age of 53 years (Table 1). MAC was the most common pathogenic NTM species, followed by *M. abscessus*. Specifically, MAC was most frequently observed in NTM-PD cases, while *M. abscessus* was most common in ENTM cases. Disseminated NTM infections were primarily caused by MAC (Table 1).

### 3.2. Different Characteristics with NTM-PD and ENTM

A total of 452 patients (340 with NTM-PD and 112 with ENTM) were included in the study. Compared to ENTM patients, those with NTM-PD were older (median age: 55 vs. 48 years, *p* = 0.001) and had a lower body mass index (BMI) (20.0 vs. 22.0 kg/m^2^, *p* = 0.015). Regarding comorbidities, pre-existing structural lung diseases—including bronchiectasis, a history of PTB, and chronic obstructive pulmonary disease (COPD)—were significantly more prevalent in NTM-PD patients. Conversely, diabetes and human immunodeficiency virus (HIV) infection were more common among ENTM patients. Given that BMI had 51.3% missing values, multiple imputation using the MICE algorithm (five imputations, 20 iterations) was performed to handle the missing data. Univariate logistic regression identified BMI, bronchiectasis, PTB history, COPD, diabetes, and HIV infection as potential predictors of NTM-PD (all *p* < 0.05). After adjusting for confounders, bronchiectasis (adjusted OR = 24.86, 95% CI: 7.58–152.32, *p* < 0.001) and a history of PTB (adjusted OR = 7.58, 95% CI: 3.22–22.08, *p* < 0.001) remained the strongest independent risk factors for NTM-PD. In contrast, higher BMI and diabetes were significantly associated with a lower risk of pulmonary infection. Although significant in univariate analysis, COPD and HIV infection did not retain statistical significance after multivariate adjustment (Table 2).

### 3.3. Clinical Differences and Differential Diagnosis Between NTM-PD and PTB

Initially, 218 patients with PTB were misdiagnosed as having NTM-PD. Of these, 86 (39.4%) were female, and the median age was 49 years. Among the 340 patients diagnosed with NTM-PD, 13 were found to have co-infection with both NTM and TB. The remaining 327 patients with purely NTM-PD were included in the subsequent comparative analysis against the PTB group.

#### 3.3.1. Different Clinical Characteristics

A total of 545 patients (327 with NTM-PD and 218 with PTB) were included in this analysis. Missing BMI data (45.7%) were handled using multiple imputation (MICE, 5 imputations, 20 iterations). Compared to PTB patients, those with NTM-PD were older (median age: 55 vs. 49 years, *p* = 0.001), had lower BMI (19.6 vs. 21.3 kg/m^2^, *p* < 0.001), and were more likely to be female. Regarding comorbidities, NTM-PD patients had a significantly higher prevalence of bronchiectasis and COPD, whereas PTB patients were more likely to have diabetes, HIV infection and chronic kidney disease (CKD). After multivariate adjustment, male sex, HIV infection, CKD and higher BMI were significantly associated with a lower likelihood of NTM-PD (i.e., favoring PTB). Conversely, COPD and bronchiectasis were independent risk factors for NTM-PD. Disease duration and the presence of symptoms such as fever, hemoptysis, and weight loss were key factors distinguishing the two conditions (Table 3).

Chest computed tomography (CT) scans at the time of diagnosis were available for 491 of the 545 patients, including 288 patients with NTM-PD and 203 patients with PTB. Although bilateral lung involvement was common in both NTM-PD and PTB, no significant difference was observed in the specific distribution patterns of lung lesions between the two groups. Nodule and patchy opacities represented the predominant imaging features observed in both diseases. However, bronchiectasis and mucus plug were more common in NTM-PD patients, whereas PTB was characterized by pleural thickening, lymphadenopathy and pleural effusion (Table S1). Laboratory parameters had a high proportion of missing values; therefore, analyses of these variables were limited to univariate comparisons using available data, without multiple imputation. NTM-PD is characterized by an elevated monocyte count and hypoproteinemia, while T-cell immune function remains relatively preserved. In contrast, patients with PTB are more prone to anemia and renal dysfunction, accompanied by a more pronounced impairment of T-cell immunity (Table S2).

#### 3.3.2. Different Anti-Mycobacterial Drug Susceptibility

The drug susceptibility profiles of clinical isolates from NTM-PD and PTB exhibit significant differences, providing crucial microbiological evidence for clinical treatment (Table S3). First-line anti-tuberculosis drugs are largely ineffective against NTM: NTM-PD shows extremely high resistance to isoniazid, while its susceptibility to rifamycin and ethambutol is considerably lower than that of PTB strains. In contrast, macrolides and amikacin maintain high activity against NTM-PD, with susceptibility rates significantly higher than those observed in the PTB group, establishing their central role in NTM-PD treatment regimens. Furthermore, fluoroquinolones also demonstrate relatively good antibacterial activity against NTM-PD (Figure 2C).

### 3.4. Predictive Model Development

**Figure 3:**
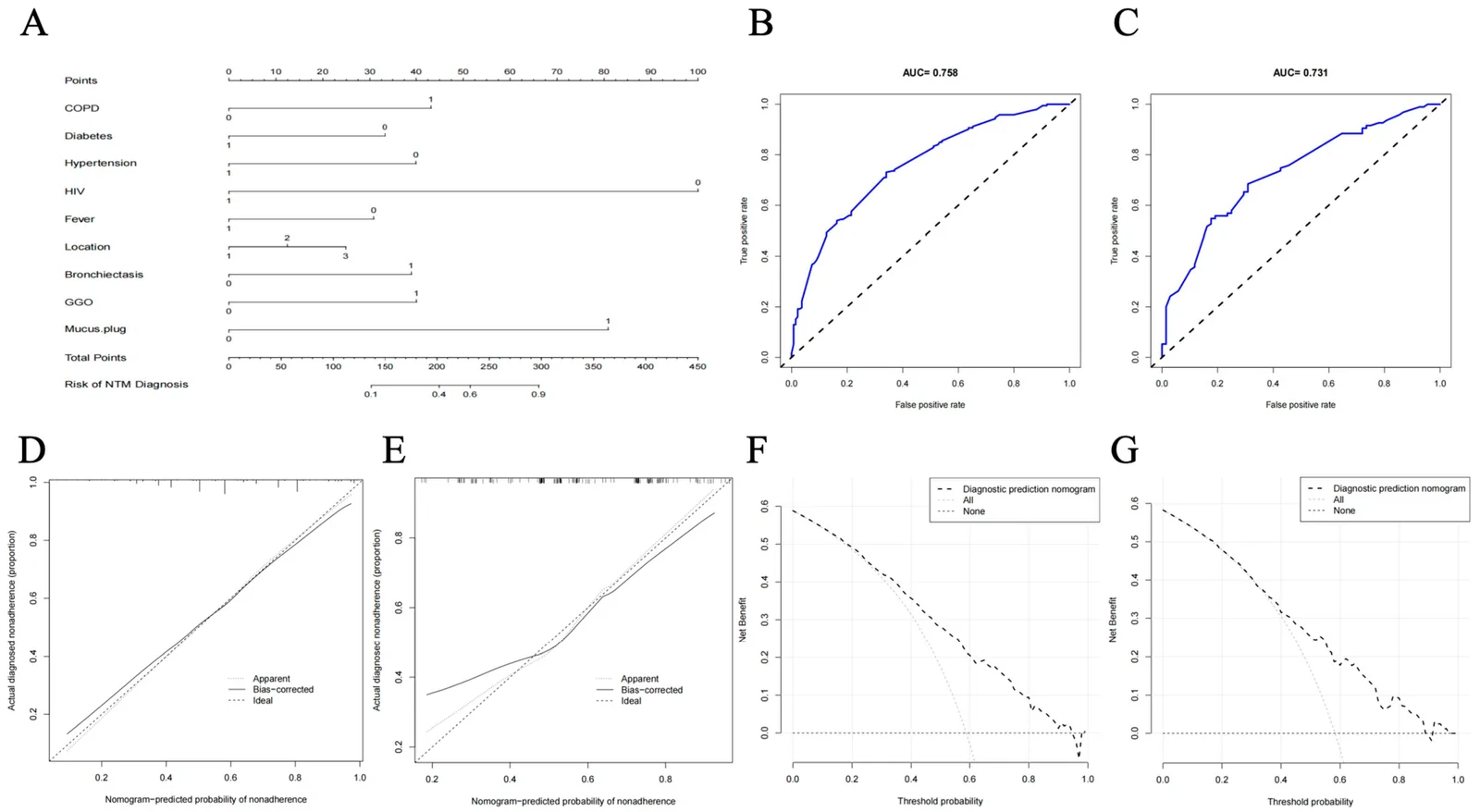
Development and performance evaluation of the NTM-PD and PTB differential diagnosis prediction model. (**A**) Nomogram of the prediction model. To use the nomogram, locate the patient’s value for each variable, draw a vertical line upward to the “Points” axis to determine the corresponding score, sum all scores to obtain the “Total Points,” and then draw a vertical line downward from the “Total Points” axis to the “Risk of NTM Diagnosis” axis to read the predicted probability of NTM. (**B**,**C**) Receiver operating characteristic (ROC) curves of the model in the (**B**) training cohort (AUC = 0.758) and (**C**) validation cohort (AUC = 0.731). (**D**,**E**) Calibration curves of the model in the (**D**) training and (**E**) validation cohorts. The dashed diagonal line represents the ideal reference, the dotted line represents the apparent accuracy, and the solid line represents the bias-corrected performance. (**F**,**G**) Decision curve analysis (DCA) of the model in the (**F**) training and (**G**) validation cohorts. The black dashed line represents the net benefit of using the present prediction model; the light gray dashed line represents the net benefit of the “treat all as NTM” strategy; the dark gray dashed line represents the net benefit of the “treat none as NTM” strategy. Abbreviations: NTM-PD: nontuberculous mycobacterial pulmonary diseases; PTB: pulmonary tuberculosis; COPD, chronic obstructive pulmonary disease; HIV, human immunodeficiency virus; GGO: ground-glass opacity; AUC: area under curve.

We developed a differential diagnostic model to distinguish NTM-PD from PTB based on clinical characteristics and imaging findings. The LASSO algorithm reduced 39 features to 22 potential predictors, which were then incorporated into a multivariate logistic regression model (Table S4). The final prediction model, constructed using variables with *p* < 0.05, included nine predictors: COPD, diabetes, hypertension, HIV, fever, lesion location, bronchiectasis, ground-glass opacity, and mucus plug (Table 4). A nomogram was developed to visualize the model (Figure 3A), and a corresponding scoring system was created to facilitate clinical application (Table S5). The model demonstrated moderate to good discriminative ability, with an AUC of 0.758 (95% CI: 0.706–0.810) in the training cohort and 0.731 (95% CI: 0.654–0.807) in the validation cohort (Figure 3B,C). Sensitivity and specificity were 0.731 and 0.659 in the training cohort, and 0.684 and 0.691 in the validation cohort, respectively (Table S6). Calibration curves showed acceptable agreement between predicted probabilities and observed outcomes in both cohorts (Figure 3D,E). The C-index was 0.632 (95% CI: 0.604–0.657) for the training cohort and 0.619 (95% CI: 0.572–0.655) for the validation cohort. Furthermore, decision curve analysis revealed that the model provided a positive net benefit across a range of threshold probabilities (Figure 3F,G). It should be noted that laboratory parameters were not included in the final predictive model due to a high proportion of missing values, which precluded their reliable incorporation into the regression analysis.

## 4. Discussion

This study systematically analyzed the distinct epidemiological characteristics of patients with pulmonary versus extrapulmonary NTM infections, compared the clinical, radiological, laboratory, and antimicrobial susceptibility profiles between NTM-PD and PTB patients, and developed an early clinical-radiological predictive model to distinguish NTM-PD from PTB.

Our analysis revealed that patients with NTM-PD were significantly older and had a lower BMI compared to those with ENTM. These differences may reflect age-related immune senescence and increased susceptibility to chronic respiratory conditions. Notably, a prospective Korean study confirmed that underweight status is an independent risk factor for NTM-PD, supporting our observation that lower BMI is more characteristic of pulmonary involvement [16]. Consistent with previous reports, NTM-PD was strongly associated with structural lung diseases, including bronchiectasis, prior PTB, and COPD [3,17]. After multivariate adjustment, bronchiectasis (OR = 24.86) and PTB history (OR = 7.58) remained the strongest independent risk factors for pulmonary NTM disease. Interestingly, while diabetes has traditionally been considered a risk factor for NTM-PD, our study found that diabetes was significantly more prevalent in ENTM patients [18]. We propose a “temporal dynamic” hypothesis to explain this apparent paradox: prior to infection, diabetes may facilitate pulmonary colonization through local immune impairment, whereas once infection is established, its systemic immunosuppressive effects may promote extrapulmonary dissemination. Additionally, HIV infection was more common in ENTM patients, underscoring the critical role of immunosuppression in facilitating extrapulmonary NTM disease. Regarding pathogen distribution, MAC was predominant in NTM-PD, while *M. abscessus* was more frequently isolated from ENTM cases, consistent with East Asian epidemiological data and suggesting species-specific tissue tropism [19].

In the comparative analysis between NTM-PD and PTB, we observed that NTM-PD patients were more likely to be female and older, whereas PTB predominated in males, consistent with previous reports [20,21]. Multivariate analysis identified COPD (OR = 1.97) and bronchiectasis (OR = 1.51) as independent risk factors for NTM-PD, while male sex (OR = 0.52), HIV infection (OR = 0.22), CKD (OR = 0.32), and higher BMI (OR = 0.93) were significantly associated with PTB.

Radiologically, bronchiectasis and mucus plugs were more common in NTM-PD, whereas pleural thickening, lymphadenopathy, and pleural effusion were characteristic of PTB, providing valuable clues for clinical differentiation. Tuberculous pleural effusion (TPE) is typically characterized as a lymphocyte-predominant exudate. However, recent evidence increasingly shows that tuberculosis can also present as neutrophil-predominant tuberculous pleural effusion (NP-TPE), which poses significant diagnostic challenges in clinical practice [22,23]. Multiple retrospective studies have reported that NP-TPE accounts for approximately 10% of all TPE cases. Compared to lymphocyte-predominant TPE, patients with NP-TPE tend to exhibit more intense pleural inflammation, as indicated by higher pleural fluid lactate dehydrogenase and adenosine deaminase levels, along with lower glucose and pH values. Additionally, thoracoscopic findings in NP-TPE more frequently reveal purulent material, caseous necrosis, and inflammatory changes [23,24]. Notably, NP-TPE is associated with lower rates of granuloma formation and positive cultures from biopsy tissues; however, some patients may transition to lymphocyte predominance during the disease course, suggesting that NP-TPE may represent either an early or a more severe phase of tuberculous pleurisy. Clinicians should remain vigilant in differentiating NP-TPE from parapneumonic effusions and consider prompt thoracoscopic biopsy to improve diagnostic yield [22,24]. Although the present study evaluated the prevalence of calcification and pleural thickening in patients with NTM-PD and PTB, the specific incidence of pleural calcification was not analyzed separately. The differential diagnosis of pleural calcification primarily includes malignant pleural mesothelioma, pleural metastases, and chronic pleuritis [25]. Tuberculous pleuritis is one of the most common causes of chronic pleural inflammation, which can progress to pleural thickening and eventual calcification over time [26]. However, pleural calcification is rarely observed as a typical feature of active NTM-PD. Therefore, in clinical practice, the identification of pleural calcification should prompt careful consideration of prior tuberculous infection, especially in regions with a high tuberculosis burden.

Laboratory findings revealed distinct immunopathological profiles: NTM-PD was characterized by elevated monocyte counts and hypoproteinemia, with relatively preserved T-cell function, suggesting a localized chronic inflammatory response with ineffective granuloma formation. In contrast, PTB exhibited more pronounced T-cell exhaustion, accompanied by anemia and renal dysfunction, reflecting systemic immune dysregulation driven by a highly virulent pathogen. Of clinical importance is the challenge of distinguishing PTB from NTM-PD in patients with positive acid-fast bacilli (AFB) smears. According to a recent multicenter study, among AFB smear-positive patients, older age, hemoptysis, dyspnea, bronchiectasis, and a history of COPD were more frequently associated with NTM-PD, whereas fever, tree-in-bud opacities, satellite nodules, and strongly positive purified protein derivative (PPD) reactions were more characteristic of PTB [27]. Nevertheless, the discriminatory power of these clinical and radiological features is limited, and considerable overlap exists between the two conditions; consequently, microbiological identification remains essential for a definitive diagnosis.

In terms of drug susceptibility, NTM demonstrated widespread resistance to conventional anti-tuberculosis drugs, consistent with their known intrinsic resistance [28]. This finding further underscores the urgency of accurately distinguishing between NTM-PD and PTB to avoid ineffective treatments and adverse drug reactions. The antimicrobial susceptibility analysis is limited by the relatively small number of isolates tested for certain agents. Additionally, pooled analyses across NTM species may obscure species-specific resistance patterns, particularly given the known differences between MAC and *M. abscessus*. Therefore, further studies with species-stratified susceptibility analyses are warranted in the future.

Based on these differences, this study developed a diagnostic model incorporating nine readily available clinical and radiological predictors, which demonstrated moderate discriminative ability in both the training cohort (AUC = 0.758, 95% CI: 0.706–0.810) and the validation cohort (AUC = 0.731, 95% CI: 0.654–0.807). Calibration curves showed acceptable agreement between predicted and observed outcomes, and decision curve analysis suggested a potential net clinical benefit across a range of threshold probabilities. The variables included in the model are routinely available in clinical practice, which may facilitate its implementation in primary care settings as a preliminary screening tool to aid in the early differentiation of NTM-PD from PTB before microbiological confirmation. However, given the model’s moderate performance, particularly in the validation cohort, its clinical utility should be interpreted with caution, as it is not intended to replace microbiological diagnosis. Further prospective studies with larger sample sizes and external validation are warranted to confirm its generalizability and refine its predictive accuracy before routine clinical application.

This study has several limitations. First, as a single-center retrospective study spanning 16 years, the results may be subject to selection bias and time-related confounding due to advances in diagnostic techniques, imaging, treatment, and diagnostic criteria over this period. Second, the NTM cohort includes multiple species with variable virulence, radiological features, disease progression, and drug susceptibility. However, species-stratified analyses were not feasible due to limited subgroup sample sizes, and all NTM cases were analyzed as a homogeneous group, which may obscure clinically significant differences. Third, the model demonstrated moderate discriminative ability in the training cohort (AUC = 0.758), with decreased performance in the validation cohort (AUC = 0.731), suggesting some overfitting and indicating potential for improvement through incorporation of multidimensional data. Finally, the impact of model-guided diagnostic strategies on long-term patient outcomes has not been evaluated. Future multicenter prospective studies with external validation are needed to address these limitations.

## 5. Conclusions

In conclusion, this study systematically characterized the distinct epidemiological, clinical, radiological, and microbiological profiles of NTM-PD compared to ENTM and PTB. We identified bronchiectasis and a prior history of PTB as the strongest independent risk factors for pulmonary NTM disease, while HIV infection and diabetes were more closely associated with extrapulmonary involvement. Furthermore, we developed and internally validated a practical clinical-radiological nomogram incorporating nine readily available predictors (COPD, diabetes, hypertension, HIV, fever, lesion location, bronchiectasis, ground-glass opacity, and mucus plug) to facilitate early differentiation between NTM-PD and PTB. The model demonstrated moderate discriminative ability (AUC: 0.758 in the training cohort and 0.731 in the validation cohort) and provided a net clinical benefit across a range of threshold probabilities, offering a useful preliminary screening tool in settings where microbiological confirmation is not immediately accessible. However, given its moderate performance and the limitations inherent in a single-center retrospective design, this nomogram should be viewed as a complementary aid rather than a replacement for pathogen-based diagnosis. Future multicenter prospective studies with external validation are essential to confirm its generalizability and refine its predictive accuracy before widespread clinical implementation.

## Figures and Tables

**Table 1:** Baseline demographic and clinical characteristics.

Characteristics	Overall (*N* = 473)	NTM-PD (*N* = 340)	ENTM (*N* = 112)	Disseminated NTM Disease (*N* = 21)
**Gender, Female, *n* (%)**	267 (56.4)	187 (55.0)	71 (63.4)	9 (42.8)
**Age, y [Median, IQR]**	53, 25	55, 21	48, 24	56, 34
**BMI, kg/m^2^ [Median, IQR]**	20.0, 4.85	20.0, 4.5	22.0, 4.6	19.5, 6.75
**Smoking, *n* (%)**	120 (25.4)	86 (25.3)	29 (25.9)	5 (23.8)
**Drinking, *n* (%)**	58 (12.3)	42 (12.4)	12 (10.7)	4 (19.0)
**Malignancy, *n* (%)**	47 (9.9)	34 (10.0)	9 (8.0)	4 (19.0)
**Comorbidity, *n* (%)**				
COPD	44 (9.3)	43 (12.6)	1 (0.9)	0 (0.0)
PTB	102 (21.6)	93 (27.4)	5 (4.5)	4 (19.0)
Bronchiectasis	124 (26.2)	122 (35.9)	2 (1.8)	0 (0.0)
Asthma	8 (1.7)	7 (2.1)	1 (0.9)	0 (0.0)
DM	35 (7.4)	18 (5.3)	15 (13.4)	2 (9.5)
Hypertension	39 (8.2)	31 (9.1)	7 (6.3)	1 (4.8)
AD/CTD	46 (9.7)	28 (8.2)	15 (13.4)	3 (14.3)
Chronic liver disease	66 (14.0)	46 (13.5)	14 (12.5)	6 (28.6)
CKD	17 (3.6)	10 (2.9)	4 (3.6)	3 (14.3)
HIV infection	19 (4.0)	6 (1.8)	7 (6.3)	6 (28.6)
**Etiological Types, *n* (%)**				
Unclassified	63 (13.3)	23 (6.8)	39 (34.8)	1 (4.8)
**SGM**				
*M. avium* complex	165 (34.9)	138 (40.6)	13 (11.6)	14 (66.7)
*M. kansasii*	19 (4.0)	17 (5.0)	2 (1.9)	0 (0.0)
*M. chimaera*	1 (0.2)	1 (0.3)	0 (0.0)	0 (0.0)
*M. marinum*	12 (2.5)	0 (0.0)	12 (10.7)	0 (0.0)
*M. marseillense*	5 (1.1)	2 (0.6)	3 (2.7)	0 (0.0)
*M. gordonae*	11 (2.3)	9 (2.6)	1 (0.9)	1 (4.8)
Others	27 (5.7)	17 (5.0)	9 (8.0)	1 (4.8)
**RGM**				
*M. abscessus*	150 (31.7)	124 (36.5)	24 (21.4)	2 (9.5)
*M. chelonae*	23 (4.9)	18 (5.3)	4 (3.7)	1 (4.8)
*M. fortuitum*	11 (2.3)	4 (1.2)	5 (4.5)	2 (9.5)
Others	9 (1.9)	5 (1.5)	4 (3.7)	0 (0.0)
**Multiple**	24 (5.1)	18 (5.3)	4 (3.7)	2 (9.5)

Abbreviation: NTM-PD, nontuberculous mycobacterial pulmonary disease; ENTM, extrapulmonary nontuberculous mycobacteria disease; NTM, nontuberculous mycobacteria; IQR, interquartile range; BMI, body mass index; COPD, chronic obstructive pulmonary disease; PTB, pulmonary tuberculosis; DM, diabetes mellitus; AD, autoimmune diseases; CTD, connective tissue disorder; CKD, chronic kidney disease; HIV, human immunodeficiency virus; RGM, rapidly growing non-tuberculous mycobacteria; SGM, slowly growing non-tuberculous mycobacteria. Italicized symbols (*n*) denote statistical variables and are used in accordance with standard conventions.

**Table 2:** Different demographic and clinical characteristics with NTM-PD and ENTM.

Characteristics	Overall (*N* = 452)	NTM-PD (*N* = 340)	ENTM (*N* = 112)	*p* Value	Univariate OR (95% CI)	Multivariate Adjusted OR (95% CI)
**Gender, Female, *n* (%)**	258 (57.1)	187 (55.0)	71 (63.4)	*0.1481*	1.42 (0.91–2.20)	-
**Age, y [Median, IQR]**	53, 24	55, 21	48, 24	0.0006 ***	1.02 (1.01–1.04)	-
**BMI, kg/m^2^ [Median, IQR]**	20.0, 4.7	20.0, 4.5	22.0, 4.6	0.0146 *	0.88 (0.82–0.94) ***	0.89 (0.83–0.96) **
**Smoking, *n* (%)**	115 (25.4)	86 (25.3)	29 (25.9)	*0.9991*	0.97 (0.59–1.58)	-
**Drinking, *n* (%)**	54 (11.9)	42 (12.4)	12 (10.7)	*0.7674*	1.17 (0.59–2.32)	-
**Malignancy, *n* (%)**	43 (9.5)	34 (10.0)	9 (8.0)	*0.668* *0*	1.27 (0.59–2.74)	-
**Comorbidity, *n* (%)**						
COPD	44 (9.7)	43 (12.6)	1 (0.9)	0.0005 ***	16.07 (2.19–118.10) **	5.75 (0.82–116.33)
PTB	98 (21.7)	93 (27.4)	5 (4.5)	<0.0001 ****	8.06 (3.19–20.38) ***	7.58 (3.22–22.08) ****
Bronchiectasis	124 (27.4)	122 (35.9)	2 (1.8)	<0.0001 ****	30.78 (7.47–126.78) ***	24.86 (7.58–152.32) ****
Asthma	8 (1.8)	7 (2.1)	1 (0.9)	*0.6858*	2.33 (0.28–19.17)	-
DM	33 (7.3)	18 (5.3)	15 (13.4)	0.0081 **	0.36 (0.18–0.74) **	0.37 (0.16–0.83) *
Hypertension	38 (8.4)	31 (9.1)	7 (6.3)	*0.4519*	1.50 (0.64–3.52)	-
AD/CTD	43 (9.5)	28 (8.2)	15 (13.4)	*0.1533*	0.58 (0.30–1.13)	-
Chronic liver disease	60 (13.3)	46 (13.5)	14 (12.5)	*0.9061*	1.10 (0.58–2.08)	-
CKD	14 (3.1)	10 (2.9)	4 (3.6)	*0.7553*	0.82 (0.25–2.66)	-
HIV infection	13 (2.9)	6 (1.8)	7 (6.3)	0.0215 *	0.27 (0.09–0.82) *	0.31 (0.07–1.13)

Abbreviations: NTM-PD, nontuberculous mycobacterial pulmonary disease; ENTM, Extrapulmonary nontuberculous mycobacteria disease; IQR, interquartile range; BMI, body mass index (BMI was imputed using multiple imputation); COPD, chronic obstructive pulmonary disease; PTB, pulmonary tuberculosis; DM, diabetes mellitus; AD, autoimmune diseases; CTD, connective tissue disorder; CKD, chronic kidney disease; HIV, human immunodeficiency virus; OR, odds ratio; CI, confidence interval. *p* value significance: * *p* < 0.05; ** *p* < 0.01; *** *p* < 0.001; **** *p* < 0.0001. Italicized symbols (*p*, *n*) denote statistical variables and are used in accordance with standard conventions.

**Table 3:** Clinical characteristic differences between NTM-PD and PTB.

Characteristics	Overall (*N* = 545)	NTM-PD (*N* = 327)	PTB (*N* = 218)	*p* Value	Univariate OR (95% CI)	Multivariate Adjusted OR (95% CI)
**Gender, Female, *n* (%)**	269 (49.4)	183 (56.0)	86 (39.4)	0.0002 ***	0.51 (0.36–0.73) ***	0.52 (0.36–0.75) ***
**Age, y [Median, IQR]**	53, 26	55, 20	49, 29	0.0013 **	1.02 (1.01–1.03)	
**BMI, kg/m^2^ [Median, IQR]**	20.2, 4.5	19.6, 4.5	21.3, 4.2	<0.0001 ****	0.88 (0.84–0.93) ***	0.93 (0.88–0.99) *
**Smoking, *n* (%)**	142 (26.1)	82 (25.1)	60 (27.5)	*0.5907*	0.88 (0.60–1.30)	
**Drinking, *n* (%)**	74 (13.7)	38 (11.6)	36 (16.5)	*0.1321*	0.66 (0.41–1.09)	
**Malignancy, *n* (%)**	46 (8.4)	32 (9.8)	14 (6.4)	*0.2199*	1.58 (0.82–3.04)	
**Comorbidity, *n* (%)**						
COPD	56 (10.3)	41 (12.5)	15 (6.9)	0.0469 *	1.94 (1.05–3.60) *	1.97 (1.03–3.94) *
Bronchiectasis	176 (32.3)	121 (37.0)	55 (25.2)	0.0053 **	1.74 (1.19–2.54) **	1.51 (1.01–2.27) *
Asthma	8 (1.5)	7 (2.1)	1 (0.5)	*0.1532*	4.75 (0.58–38.86)	-
DM	40 (7.3)	17 (5.2)	23 (10.6)	0.0293 *	0.46 (0.24–0.89) *	0.66 (0.32–1.32)
Hypertension	56 (10.3)	29 (8.9)	27 (12.4)	*0.2377*	0.69 (0.40–1.20)	-
AD/CTD	39 (7.2)	26 (8.0)	13 (6.0)	*0.4762*	1.36 (0.68–2.71)	-
Chronic liver disease	76 (13.9)	45 (13.8)	31 (14.2)	*0.9799*	0.96 (0.59–1.58)	-
CKD	24 (4.4)	9 (2.8)	15 (6.9)	0.0368 *	0.38 (0.16–0.89) *	0.32 (0.12–0.77) *
HIV infection	18 (3.3)	5 (1.5)	13 (6.0)	0.0095 **	0.24 (0.09–0.70) **	0.22 (0.07–0.61) **
**Symptoms, *n* (%)**						
Duration (≥2 month)	427 (78.3)	268 (82.0)	159 (72.9)	0.0164 *	-	-
Cough	442 (81.1)	271 (82.4)	171 (78.4)	*0.2365*	-	-
Fever	126 (23.1)	55 (16.8)	71 (32.6)	<0.0001 ****	-	-
Dyspnea	129 (23.7)	75 (22.9)	54 (24.8)	*0.6959*	-	-
hemoptysis	120 (22.0)	86 (26.3)	34 (15.6)	0.0044 **	-	-
Fatigue	54 (9.9)	26 (8.0)	28 (12.8)	*0.0842*	-	-
Weight loss	45 (8.3)	17 (5.2)	28 (12.8)	0.0025 **	-	-
Chest pain	46 (8.4)	29 (8.9)	27 (12.4)	*0.2377*	-	-

Abbreviations: NTM-PD, nontuberculous mycobacteria pulmonary disease; PTB, pulmonary tuberculosis; IQR, interquartile range; BMI, body mass index (BMI was imputed using multiple imputation); COPD, chronic obstructive pulmonary disease; DM, diabetes mellitus; AD, autoimmune diseases; CTD, connective tissue disorder; CKD, chronic kidney disease; HIV, human immunodeficiency virus; OR, odds ratio; CI, confidence interval. *p* value significance: * *p* < 0.05; ** *p* < 0.01; *** *p* < 0.001; **** *p* < 0.0001. Italicized symbols (*p*, *n*) denote statistical variables and are used in accordance with standard conventions.

**Table 4:** Multivariable logistic analyses of prediction factors.

Intercept and Variable	Prediction Model		
	β	Odds Ratio (95% CI)	*p* Value
Intercept	−0.6916	0.501(0.219–1.127)	0.0961
COPD	1.1766	3.243 (1.276–9.116)	0.0179 *
Diabetes	−0.9074	0.404 (0.145–1.058)	0.0702 *
Hypertension	−1.0890	0.337 (0.138–0.782)	0.0129 *
HIV infection	−2.7267	0.065 (0.005–0.411)	0.0090 **
Fever	−0.8397	0.432 (0.234–70.781)	0.0060 **
Location	0.3392	1.404 (1.011–1.956)	0.0434 *
Bronchiectasis	1.0625	2.893(1.712–4.979)	<0.0001 ****
GGO	1.0919	2.980(1.162–8.356)	0.0285 *
Mucus plug	2.2052	9.072(2.201–72.030)	0.0095 **

Abbreviations: COPD, chronic obstructive pulmonary disease; HIV, human immunodeficiency virus; GGO, ground-glass opacity; CI, confidence interval. *p* Value significance: * *p* < 0.05; ** *p* < 0.01; **** *p* < 0.0001. Italicized symbols (*p*) denote statistical variables and are used in accordance with standard conventions.

## Data Availability

The datasets used and analyzed during the current study are available from the corresponding author on reasonable request.

## Supplementary Materials

The following supporting information can be downloaded at: https://www.mdpi.com/article/10.3390/jcm15176796/s1.

## Abbreviations

The following abbreviations are used in this manuscript:

NTM	Nontuberculous mycobacteria
MAC	Mycobacterium avium-intracellulare complex
NTM-PD	NTM pulmonary diseases
PTB	pulmonary tuberculosis
ATS/IDSA	American Thoracic Society/Infectious Diseases Society of America
WHO	World Health Organization
MIC	minimum inhibitory concentration
CLSI	Clinical and Laboratory Standards Institute
SD	standard deviation
IQR	interquartile range
LASSO	Least Absolute Shrinkage and Selection Operator
AUC	area under curve
DCA	decision curve analysis
ENTM	extrapulmonary NTM
BMI	body mass index
COPD	chronic obstructive pulmonary disease
HIV	human immunodeficiency virus
AFB	acid-fast bacilli
PPD	purified protein derivative
