# Supplementary material for: Differentiating Nontuberculous Mycobacterial Lung Disease from Pulmonary Tuberculosis: A Retrospective Study on Clinical Features and a Diagnostic Model
Source: J Clin Med. 2026 Sep 2;15(17):6796. doi: 10.3390/jcm15176796 (PMC13566219; doi:10.3390/jcm15176796)
Supplement: Supplementary file 1 [file jcm-15-06796-s001.zip › jcm-4415886-supplementary.pdf]

Figure S1. Variable Selection Process of the LASSO Regression Model.

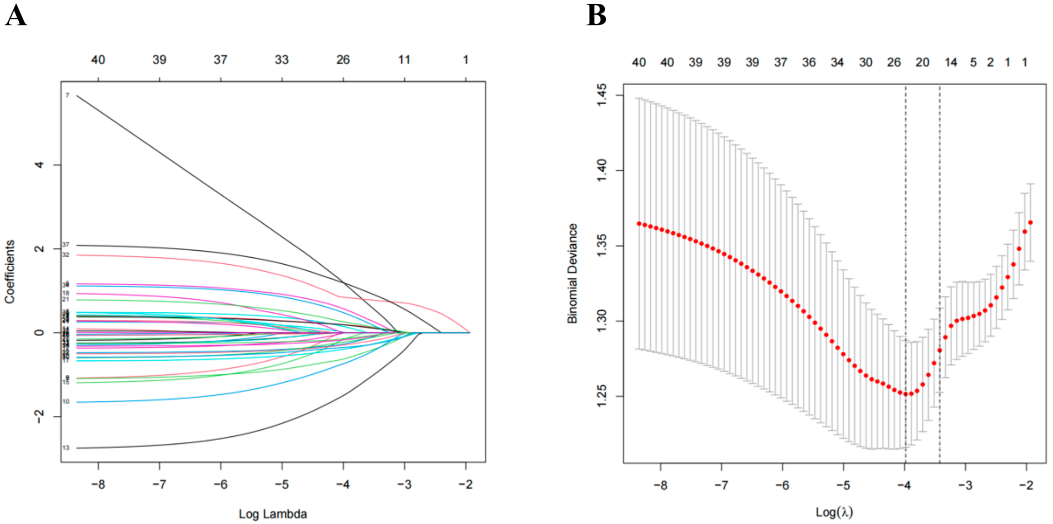

(A): Shows the coefficient paths of the LASSO regression model. The x-axis represents the logarithm of the regularization parameter (Log Lambda), while the y-axis shows the standardized coefficients of the predictor variables. Each curve corresponds to a different predictor variable. As the Lambda value increases (moving from left to right), the penalty strength intensifies, causing more coefficients to shrink toward zero, effectively excluding those variables from the model.

(B): Shows the cross-validation error curve of the LASSO regression. The x-axis represents the logarithm of the Lambda values, while the y-axis shows the binomial deviance of the model, which quantifies model fit (lower values indicate a better fit). Each point on the curve represents the mean deviance obtained through cross-validation at different Lambda values, with error bars indicating the standard error of the deviance. The left vertical dashed line marks the optimal Lambda value that minimizes the cross-validation error. The right vertical dashed line indicates the Lambda value corresponding to the most parsimonious model (i.e., the model with the fewest variables) within one standard error of the optimal model.

Table S1. Imaging differences between NTM-PD and PTB.

Characteristics	Overall (N = 491)	NTM-PD (N = 288)	PTB (N = 203)	*P* value
**Location**, n (%)				0.2495
Both	251 (51.1)	156 (54.2)	95 (46.8)	0.1293
Right	152 (31.0)	82 (28.5)	70 (34.5)	0.1870
Light	88 (17.9)	50 (17.4)	38 (18.7)	0.7895
**Imaging features**, n (%)				
Patchy opacities	317 (64.6)	191 (66.3)	126 (62.1)	0.3822
Nodule	421 (85.7)	244 (84.7)	177 (87.2)	0.5223
Mass	56 (11.4)	29 (10.1)	27 (13.3)	0.3345
Cavity	130 (26.5)	70 (24.3)	60 (29.6)	0.2321
Consolidation	120 (24.4)	74 (25.7)	46 (22.7)	0.5068
Bronchiectasis	215 (43.8)	161 (55.9)	54 (26.6)	***<0.0001*******
Calcification	100 (20.4)	52 (18.1)	48 (23.6)	0.1613
Ground-glass opacity	43 (8.8)	30 (10.4)	13 (6.4)	0.1655
Tree-in-bud	6 (1.2)	5 (1.7)	1 (0.5)	0.4083
Reticulation	128 (26.1)	69 (24.0)	59 (29.1)	0.2441
Mucus plug	44 (9.0)	40 (13.9)	4 (2.0)	***<0.0001*******
Pleural thickening	186 (37.9)	95 (33.0)	91 (44.8)	***0.0102****
Lymphadenopathy	187 (38.1)	96 (33.3)	91 (44.8)	***0.0128****
Pleural effusion	48 (9.8)	18 (6.3)	30 (14.8)	***0.0029*****

Abbreviations: NTM-PD: nontuberculous mycobacterial pulmonary diseases; PTB: pulmonary tuberculosis.

Table S2. Laboratory tests' differences between NTM-PD and PTB.

Characteristics	Overall	NTM-PD	PTB	*P* value
**Complete Blood Count**, N (%)				
RBC				***0.0055****
High	25 (5.4)	15 (5.9)	10 (4.8)	
Normal	337 (72.9)	199 (78.0)	138 (66.7)	
Low	100 (21.6)	41 (16.1)	59 (28.5)	
HGB				***0.0005*****
High	10 (2.2)	5 (2.0)	5 (2.4)	
Normal	308 (66.7)	189 (74.1)	119 (57.5)	
Low	144 (31.2)	61 (23.9)	83 (40.1)	
WBC				***0.0231****
High	42 (9.1)	20 (7.8)	22 (10.6)	
Normal	359 (77.7)	210 (82.4)	149 (72.0)	
Low	61 (13.2)	25 (9.8)	36 (17.4)	
NEUT				0.1249
High	79 (17.1)	39 (15.3)	40 (19.3)	
Normal	352 (76.2)	203 (79.6)	149 (72.0)	
Low	31 (6.7)	13 (5.1)	18 (8.7)	
LYMPH				0.3373
Normal	348 (75.3)	197 (77.3)	151 (72.9)	
Low	114 (24.7)	58 (22.7)	56 (27.1)	
MONO				***0.0062****
High	78 (16.9)	55 (21.6)	23 (11.1)	
Normal	380 (82.3)	198 (77.6)	182 (87.9)	
Low	4 (0.9)	2 (0.8)	2 (1.0)	
EOS				0.7931
High	22 (4.8)	12 (4.7)	10 (4.8)	
Normal	402 (87.0)	224 (87.8)	178 (86.0)	
Low	38 (8.2)	19 (7.5)	19 (9.2)	
**Liver Function Tests**, N (%)				

ALT				0.5130
High	55 (12.4)	32 (13.6)	23 (11.1)	
Normal	389 (87.6)	204 (86.4)	185 (88.9)	
AST				1.0000
High	48 (10.8)	26 (11.0)	22 (10.6)	
Normal	396 (89.2)	210 (89.0)	186 (89.4)	
Albumin				***0.0429****
High	4 (0.9)	4 (1.7)	0 (0.0)	
Normal	271 (62.3)	135 (57.0)	136 (65.4)	
Low	160 (36.8)	98 (41.4)	72 (34.6)	
**Renal Function Tests**, N (%)				
Cr				***0.0004******
High	22 (5.0)	11 (4.7)	11 (5.3)	
Normal	371 (83.6)	211 (89.4)	160 (76.9)	
Low	51 (11.5)	14 (5.9)	37 (17.8)	
BUN				***0.0010*****
High	43 (12.5)	24 (10.2)	19 (9.1)	
Normal	254 (73.8)	199 (84.3)	155 (74.5)	
Low	47 (13.7)	13 (5.5)	34 (16.3)	
**T Cell Tests**, N (%)				
CD4+ T cell counts				***0.0205****
Normal	70 (47.6)	52 (55.3)	18 (34.0)	
Low	77 (52.4)	42 (44.7)	35 (66.0)	
CD8+ T cell counts				0.8466
Normal	110 (77.5)	73 (78.5)	37 (75.5)	
Low	32 (22.5)	20 (21.5)	12 (24.5)	

Abbreviations: RBC: red blood cells; HGB: hemoglobin; WBC: white blood cells; NEUT: neutrophils; LYMPH: lymphocytes; MONO: monocytes; EOS: eosinophil count; ALT: alanine aminotransferase; AST: aspartate aminotransferase; Cr: creatinine; BUN: blood urea nitrogen.

Table S3. Antimicrobial susceptibility testing results.

Antibiotics	Type	Susceptible (N, %)	Intermediate (N, %)	Resistant (N, %)	MIC Breakpoint (µg/mL)	
Isoniazid	NTM-PD	3, 21.43	0, 0.00	11, 78.57	-	
Isoniazid	PTB	17, 47.22	16, 44.44	3, 8.33	<0.05	>0.2
Rifamycins	NTM-PD	37, 44.05	1, 1.19	46, 54.76	<1	>2
Rifamycins	PTB	30, 37.97	43, 54.43	6, 7.59	<0.5	>1
Amikacin	NTM-PD	95, 83.33	16, 14.04	3, 2.63	<16	>64
Amikacin	PTB	11, 57.89	1, 5.26	7, 36.84	<16	>64
Ethambutol	NTM-PD	9, 29.03	3, 9.68	19, 61.29	-	
Ethambutol	PTB	18, 62.07	9, 31.03	2, 6.90		>5
Fluoroquinolones	NTM-PD	62, 56.88	35, 32.11	12, 11.01	<1	>4
Fluoroquinolones	PTB	11, 57.89	3, 15.79	5, 26.32	<0.5	>1
Macrolides	NTM-PD	70, 63.06	24, 21.62	17, 15.32	<2	>8
Macrolides	PTB	5, 38.46	1, 7.69	7, 53.85	-	
Cefoxitin	NTM-PD	36, 39.56	27, 29.67	28, 30.77	<16	>128
Cefoxitin	PTB	4, 57.14	2, 28.57	1, 14.29	-	
Tetracyclines	NTM-PD	8, 8.42	9, 9.47	78, 82.11	<1	>8
Tetracyclines	PTB	4, 57.14	2, 28.57	1, 14.29	-	
Linezolid	NTM-PD	52, 48.60	8, 7.48	47, 43.93	<8	>32
Linezolid	PTB	3, 60.00	1, 20.00	1, 20.00	<1	>2

Abbreviations: NTM-PD: nontuberculous mycobacterial pulmonary diseases; PTB: pulmonary tuberculosis; MIC: Minimum Inhibitory Concentration.

Table S4. Multivariable logistic analyses of twenty-two potential predictors.

Intercept and variable	Prediction model			
	β	Std.Error	Z-Value	*P* value
Intercept	0.08543	0.70516	0.121	0.903573
Age	0.31799	0.21661	1.468	0.142089
Malignancy	0.57161	0.45970	1.243	0.213706
COPD	1.27673	0.53583	2.383	0.017186 **
Asthma	15.19758	628.66850	0.024	0.980714
DM	-1.12917	0.53574	-2.108	0.035059 **
Hypertension	-1.40430	0.47887	-2.933	0.003362 ***
AD/CTD	0.35125	0.51562	0.681	0.495742
Chronic liver disease	-0.39651	0.40868	-0.970	0.331941
HIV	-2.72752	1.15919	-2.353	0.018625 **
Symptoms	-0.59388	0.48412	-1.227	0.219926
Fever	-0.68497	0.33658	-2.035	0.041842 **
Hemoptysis	0.57969	0.36958	1.569	0.116764
Weight loss	-0.67024	0.52981	-1.265	0.205850
Location	0.37283	0.18068	2.063	0.039070 **
Type	-0.05243	0.14875	-0.352	0.724477
Cavity	-0.32739	0.29895	-1.095	0.273459
Bronchiectasis	1.08806	0.30685	3.546	0.000391 ***
Calcification	-0.33195	0.34850	-0.953	0.340842
Ground-glass opacity	1.14385	0.53606	2.134	0.032860 **
Mucus plug	2.00459	0.85353	2.349	0.018845 **
Pleural thickening	-0.53556	0.29236	-1.832	0.066971 *
Lymphadenopathy	-0.50754	0.29356	-1.729	0.083826 *
Pleural effusion	-0.47940	0.48244	-0.994	0.320369

Abbreviations: NTM-PD: nontuberculous mycobacterial pulmonary diseases; PTB: pulmonary tuberculosis; COPD, chronic obstructive pulmonary disease; DM, diabetes mellitus; AD, autoimmune diseases; CTD, connective tissue disorder; HIV, human immunodeficiency virus.

Table S5. A novel scoring system developed from a nomogram of the training cohort.

Intercept and variable	Scores	
	Yes	No
COPD (Yes)	43	0
Diabetes (No)	0	33
Hypertension (No)	0	40
HIV(No)	0	100
Fever (No)	0	31
Location (Bilateral)	25	0~12
Bronchiectasis (Yes)	39	0
Ground-glass opacity (Yes)	40	0
Mucus plug (Yes)	81	0

Abbreviations: COPD, chronic obstructive pulmonary disease; HIV, human immunodeficiency virus.

Table S6. Diagnostic performance of the prediction model for NTM diagnosis.

variable	Prediction model	
	**training cohort**	**validation cohort**
AUC (95%CI)	0.758 (0.706-0.810)	0.731 (0.654-0.807)
Sensitivity (95%CI)	0.731(0.617-0.803)	0.684(0.514-0.788)
Specificity (95%CI)	0.659(0.526-0.741)	0.691(0.485-0.809)
NLR (95%CI)	0.408(0.319-0.582)	0.457(0.336-0.615)
PLR (95%CI)	2.144(1.900-5.051)	2.215(1.864-5.103)

Abbreviations: AUC, area under curve; CI, confidence interval; PLR, positive likelihood ratio; NLR, negative likelihood ratio.
